# Cutting-Edge Advances in Anticancer Therapies: Insights from the Third Edition of the Special Issue “Novel Anticancer Strategies”

**DOI:** 10.3390/pharmaceutics17010054

**Published:** 2025-01-03

**Authors:** Hassan Bousbaa

**Affiliations:** UNIPRO—Oral Pathology and Rehabilitation Research Unit, University Institute of Health Sciences (IUCS), Cooperativa de Ensino Superior Politécnico e Universitário (CESPU), Rua Central de Gandra, 1317, 4585-116 Gandra, Portugal; hassan.bousbaa@iucs.cespu.pt

Cancer incidence and mortality continue to rise at an alarming rate worldwide, underscoring the urgent need for more effective therapeutic interventions. Due to the dynamic, adaptable, and complex nature of cancer progression, as well as the side effects and resistance associated with current therapies, the search for innovative drugs and drug delivery strategies remains a pressing challenge. Novel therapeutic strategies are essential to overcome the limitations of existing clinical approaches and to provide patients with more effective treatment options.

This Special Issue brings together groundbreaking research across diverse themes in cancer therapy, highlighting the innovative approaches and interdisciplinary efforts driving progress in the field of oncology. Twenty-one papers have been published, showcasing significant advancements in cancer research across various themes, ranging from metabolism-targeted therapies to cutting-edge nanotechnology and immunotherapy approaches. Each thematic area represents a critical aspect of modern oncology, underscoring the diverse strategies being developed to enhance treatment outcomes.

Metabolism and molecular pathways are pivotal in cancer progression and therapy resistance. Several studies have demonstrated the therapeutic potential of targeting these pathways. For instance, the inhibition of lactate transport using AZD3965 has shown promise in treating muscle-invasive urothelial bladder cancer by disrupting tumor metabolism [1]. Similarly, lipid metabolism has emerged as a key therapeutic focus, with Angiotensinogen (AGT) identified as a novel target in gastric cancer, particularly for patients undergoing neoadjuvant chemotherapy [2]. Metal-based therapeutics, such as ruthenium–cyclopentadienyl complexes, have also proven effective in disrupting the hallmarks of colorectal cancer [3]. Additionally, the role of the microbiome in cancer therapy is gaining traction, with short-chain fatty acids identified as critical modulators of colorectal cancer progression, offering new avenues for treatment [4].

Nanotechnology and advanced drug delivery systems are revolutionizing cancer therapy by enabling the precise targeting and minimizing systemic toxicity. pH-responsive nanoparticles, such as hyaluronic acid-conjugated cyclodextrin, have enhanced dual chemo- and CO-gas therapies [5]. Graphene nanomaterials have also demonstrated significant anticancer efficacy, namely exploiting the characteristics of the lysosomal microenvironment and the unique features of tumor cell lysosomes, as reviewed in [6]. Lipid-based and silver nanoparticles loaded with essential oils are highly effective at inducing apoptosis in cancer cells, showing superior anticancer and antimicrobial properties compared to traditional cytostatic drugs, offering a promising approach for innovative cancer therapy [7,8]. The article by Egorova et al. reviewed smart delivery systems responsive to cathepsin B activity, showing enhanced targeting and release of therapeutic agents specifically to cancer cells, thereby improving treatment efficacy and reducing side effects [9]. Photodynamic therapy is another area of innovation, particularly for melanoma and prostate cancer. Studies combining photodynamic therapy with cannabidiol or photosensitizers have shown synergistic effects in treating metastatic melanoma and prostate cancer, paving the way for more effective combination therapies [10,11,12].

Radioligand and targeted therapies are transforming treatment for specific cancers by enhancing precision and reducing side effects. Radioligands such as 177Lu-iPSMA and 177Lu-DOTATOC have been particularly effective at improving survival rates and quality of life for patients with prostate and neuroendocrine tumors [13]. Similarly, advancements in lung cancer treatment, such as combining targeted therapies with immune checkpoint inhibitors, are redefining the standard of care [14].

Combination therapies and synthetic lethality-based approaches are becoming increasingly relevant in oncology. Leveraging synthetic lethality alongside chemotherapy has been shown to enhance efficacy by exploiting cancer-specific vulnerabilities [15]. This approach has been particularly impactful in oral cancer, where multimodal strategies have improved treatment outcomes for this challenging malignancy [16].

Immunotherapy and oncolytic approaches have harnessed the body’s immune system and viruses to target cancer cells effectively. RNA interference technology, for example, has demonstrated the potential to treat papillomavirus-associated cancers by selectively knocking down oncogenes with minimal toxicity [17]. Additionally, integrating microRNA mechanisms into oncolytic viruses has enhanced their safety and efficacy, signaling a new era in virotherapy [18].

Innovations in treating hard-to-treat cancers such as glioblastoma are also at the forefront of cancer research. Novel therapeutic strategies are addressing the unique challenges posed by this aggressive brain tumor, offering hope for improved outcomes in a patient population with historically limited options [19].

Finally, preclinical studies and computational approaches are essential for advancing cancer research. Natural chalcone-based nanoparticles have shown promise in overcoming preclinical challenges in colon cancer therapy, while in silico methods are streamlining vaccine development, accelerating the transition of promising candidates into clinical use [20,21].

Together, these thematic areas underscore the multifaceted nature of cancer research, where advancements in basic science, technology, and clinical applications converge to transform the treatment landscape. Each study contributes to a deeper understanding of cancer biology and provides hope for more effective and personalized therapies.

This third volume of the Special Issue builds on the remarkable success of the previous editions, which were also dedicated to exploring cutting-edge advancements in anticancer research. The first volume, published in 2021 (https://www.mdpi.com/journal/pharmaceutics/special_issues/novel_anticancer; accessed on 18 February 2021), and the second volume, released in 2023 (https://www.mdpi.com/journal/pharmaceutics/special_issues/novel_anticancer_volume_II; accessed on 10 February 2023), both attracted a wealth of high-quality contributions, showcasing innovative research in areas such as novel drug delivery systems, the enhancement of approved anticancer agents, and the validation of new therapeutic compounds [22,23]. This third volume continues the tradition of quality, featuring original research articles and comprehensive reviews that highlight significant advances in this field. We are confident that it will serve as a valuable resource for the scientific and clinical community, fostering further exploration and collaboration in the development of novel anticancer strategies.
